# Cardiac Magnetic Resonance Imaging in Ischemic Heart Disease: A Clinical Review

**Published:** 2011-11-24

**Authors:** A. Florian, R. Jurcut, C. Ginghina, J. Bogaert

**Affiliations:** * Radiology Department, UZ Leuven, Leuven, Belgium; ** Cardiology Department, “CC Iliescu” Institute for Emergency in Cardiovascular Diseases, Bucharest, Romania; *** “Carol Davila” University of Medicine and Pharmacy, Bucharest, Romania

**Keywords:** cardiac imaging, myocardial infarction, coronary artery disease

## Abstract

Cardiac magnetic resonance imaging (MRI) has emerged as a prime player in the clinical and preclinical detection of ischemic heart disease (IHD) as well in the prognosis assessment by offering a comprehensive approach for all spectrums of coronary artery disease (CAD) patients. The aim of this review is to provide the reader a state–of–the art on how the newest cardiac MRI techniques can be used to study IHD patients.

In patients with suspected/stable CAD, functional and perfusion imaging both at rest and during vasodilatatory stress (adenosine, dypiridamole)/dobutamine stress can accurately depict ischemic myocardium secondary to significant coronary artery stenosis.

In patients with acute MI, MRI is a robust tool for differentiating and sizing the jeopardized and the infarcted myocardium by using a combination of functional, edema, perfusion and Gd contrast imaging. Moreover, important prognostic factors like myocardial salvage, the presence of microvascular obstruction (MVO), post reperfusion myocardial hemorrhage, RV involvement and infarct related complications can be assessed in the same examination. In patients with chronic ischemic cardiomyopathy, the role of the MRI extends from diagnosis by means of Gadolinium contrast scar imaging to therapy and prognosis by functional assessment and viability testing with rest and dobutamine stress imaging.

In all the circumstances mentioned, MRI derived information has been proven valuable in every day clinical decision making and prognosis assessment. Thus, MRI is becoming more and more an accepted alternative to other imaging modalities both in the acute and chronic setting.

## Introduction

Although mortality associated with ischemic heart disease (IHD) has declined in the recent decades due to the therapeutic improvements and to the prevention campaigns reducing the incidence of myocardial infarction (MI), IHD remains the leading cause of death in adults in developed countries and its prevalence will continue to increase [**1**].

In times of constrained financial budgets, the increasing prevalence of IHD will urge for rational use of diagnostic and therapeutic means. In the last 2 decades magnetic resonance imaging (MRI) has emerged as a prime player in the clinical and preclinical detection of IHD as well in the prognosis assessment [**2**].

The aim of this review is to provide the reader a state–of–the art on how the newest cardiac MRI techniques can be used to study IHD patients and to enlarge the knowledge on the complex IHD pathophysiology, with highlights on detection of obstructive coronary artery disease (CAD), on acute coronary syndromes and on chronic ischemic cardiomyopathy.

### Pathophysiological considerations

It is important to start by briefly outlining a number of different states, and their physiopathological substrates, in which acutely or chronically aggressed myocardial tissue can be found secondary to CAD.

After the acute coronary occlusion, the time frame of events in the ***ischemic cascade*** comprises the ***reversible*** changes: myocardial contractile dysfunction (seconds), intracellular ***oedema*** (20–30 minutes of sustained ischemia) followed by ***irreversible injury to myocytes*** (30–60 minutes) and ***vascular endothelial cells*** (60–90 minutes) with cellular necrosis and apoptosis. While myocardial necrosis progresses in a ***wave front*** manner from the subendocardium towards the subepicardium (3–6 hours), making transmurality time dependent, the lateral boundaries of infarction closely correspond to the initial myocardium at risk [**3**]. ***The jeopardized myocardium (myocardium at risk) ***refers to the myocardium in the perfusion territory of the involved artery distal to the critical lesion.

Urgent restoration of epicardial flow, which is the cornerstone of modern therapeutic strategies in acute MI, aims to salvage the jeopardized but viable myocardium in the myocardium at risk. Despite the beneficial effects of reperfusion, the process of cell death may continue during the first hours of reperfusion, a phenomenon called “***myocardial reperfusion injury***” [**4**]. The main feature of this complex phenomenon is the presence of ***microvascular obstruction (MVO)*** with a complete lack of tissue perfusion, despite successful epicardial recanalization.

Prolonged post–ischemic contractile dysfunction of the myocardium salvaged by reperfusion defines the “***stunned myocardium***” which may take days to weeks to normalize, though it can be reversed with inotropic agents [**5**].

Finally, the term “***hibernating myocardium***” is used to describe viable myocardium in a state of persistent but potentially reversible dysfunction secondary to a chronic coronary artery stenosis with impaired myocardial blood flow [**6**].

### Imaging Strategies in Ischemic Heart Disease

Currently available cardiac MRI techniques are able to fulfill the aims of imaging in IHD patients: on one hand, ***anatomic imaging*** with visualization of CAD and on the other hand, ***ischemia imaging*** with evaluation of the consequences of CAD to the heart, particularly myocardial perfusion and function and depiction of irreversible myocardial damage. Due to its superiority over MRI, cardiac computed tomography is used nowadays for mere visualization of coronary artery stenoses, cardiac MRI being primarily focused on the assessment of the ischemic consequences of CAD [**7–9**].

In clinical practice, by using a comprehensive MRI approach, the above information can be obtained within a single examination taking not more than 30–45 minutes.

### Functional Imaging

ECG gated and acquired during breath holds, ***dynamic cine MRI (b–SSFP) sequences*** provide a non–invasive, accurate and reproducible alternative to conventional echocardiography for ***calculating ventricular volumes and function*** and visualizing ***regional wall motion and contraction patterns***. Thus, cine MRI should be considered as a fast and robust imaging modality for both daily clinical routine and research purposes [**10, 11**]. With techniques as ***real–time non–gated cine sequences***, problems like the presence of atrial fibrillation or the incapacity for breath holding are now mostly overcome.

**Fig. 1:**
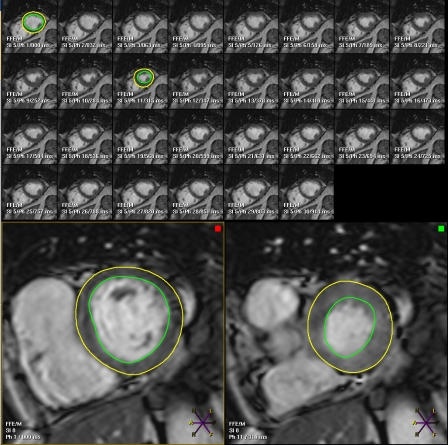
Functional analysis of short axis cine MRI. End–diastolic (ED) and end–systolic (ES) time frames are defined and then the endo– and epicardial borders are manually drawn for each slice – in this example a midventricular slice is shown.

For volumetric and functional ventricular analysis, the heart is typically studied in different cardiac imaging planes. Hereby the ventricles are completely encompassed by a set of contiguous images (usually 8 to 12), in at least one direction which is usually the short–axis. Contouring of endo– and epicardial borders of this stack of images at end-diastole and end–systole provides global functional parameters (i.e., EDV, ESV, SV, EF and myocardial mass) (**Fig. 1**).

Regional ventricular function can be assessed either qualitatively (normal, hypokinetic, akinetic, or dyskinetic) or quantitatively (relative or absolute wall thickening and wall motion). In order to have consensus between readers and imaging modalities, the standardized 17–segment model as proposed by the American Heart Association is usually used [**12**].

Further, for a more comprehensive approach of CAD patients, evaluation of cardiac function and myocardial contractility can be performed both at rest and under stress conditions (physical or pharmacological), i.e. ***ischemia/viability testing***. Because available imaging time per stress level is limited, ***fast cine MRI sequences*** are preferable in these cases. As further shown, interpretation of regional motion and contraction patterns is usually done by looking also at other types of sequences like contrast-enhanced MRI (ce–MRI) with late gadolinium enhancement which depict acute and/or healed myocardial infarction [**13**].

**Fig. 2:**
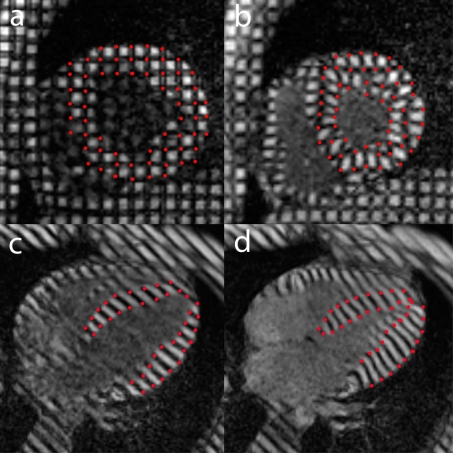
Example of MRI study with 2D tagging analysis. Tagging in cardiac short axis (**a**,**b**) and horizontal long axis (**c**,**d**), end–diastolic (left) and end–systolic time frame (right). Tracking of the grid intersections *(indicated in red)* on the short axis views, and the intersections of the tags with the endo- and epicardial border *(indicated in red)* on the long-axis views, allow analyzing the local myocardial deformation.

***Myocardial tagging MRI techniques*** non–invasively create tag or grid lines on the myocardium, allowing to analyze regional myocardial deformation 2– or 3 dimensionally throughout the cardiac cycle, and to calculate myocardial strains (**Fig. 2**).

A better characterization of the mechanisms of normal/impaired myocardial contraction is thus achieved but due to the elaborative post–processing, the clinical use of myocardial tagging MRI is currently limited [**14**].

### Myocardial Perfusion Imaging

Basic pathophysiologic principles of coronary blood flow and myocardial perfusion apply also in cardiac MRI perfusion interpretation. Briefly, in resting conditions, due to the coronary vasodilator reserve, myocardial perfusion is not altered until the coronary artery is 85–90% narrowed. In contrast, during stress the myocardium distal to less severe coronary stenosis (i.e. between 50 to 85%) may become ischemic and the coronary artery stenosis can be considered hemodynamically significant.

**Fig. 3:**
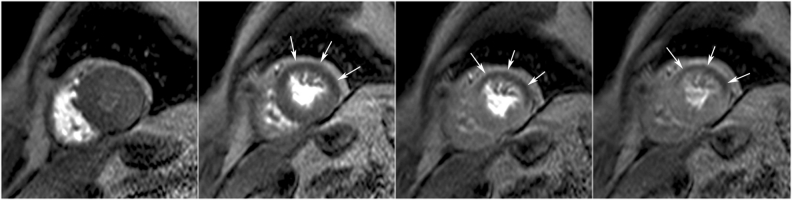
MRI stress perfusion study in a 73 old year female with suspected mid LAD coronary artery in–stent stenosis. Midventricular short–axis serial time frames of “first pass” perfusion during dipyridamole vasodilatatory stress show Gd contrast successively enhancing the right, left chambers and myocardium (*images from left to right*). A transmural perfusion defect in the anterior and lateral walls is seen (*arrows*). The patient was referred for catheterization which confirmed a 90% in–stent stenosis and was treated by PCI with drug eluting stent placement.

The most frequently used approach to assess myocardial perfusion with MRI is monitoring of the ***“first-pass”*** of contrast medium through the heart, using a bolus injection of gadolinium in combination with ***ultra-fast cine MR sequences***. Perfusion studies can be performed during resting conditions and/or during administration of a vasodilatatory agent (i.e. adenosine or dipyridamole). Whereas, normally perfused myocardium enhances homogeneously, becoming bright, hypo-or non perfused regions appear dark for a variable amount of time during/after first–pass, are most intense in the subendocardium and typically respect coronary artery perfusion territories(**Fig. 3**).

### Edema Imaging

Increased free water in the infarcted myocardium changes tissue magnetization properties (i.e. prolongs proton T1– and T2–relaxation) when compared to normal myocardium, and this change is related to the duration of ischemia [**15**]. T2–relaxation time is linearly correlated to the percentage of free water, and edema is visible on ***T2–weighted MR sequences*** in infarcted myocardium as bright areas (hyper–intense). In acute MI patients, it is accepted that the ***bright myocardium*** on T2–weighted imaging reflects the ***myocardium at risk***. Abnormalities are most evident in the acute and sub–acute phase of MI and slowly fade away due to processes of infarct healing with scar formation and resorption of infarct-related myocardial edema and inflammation [**16**]. Thus, in addition T2–weighted imaging distinguishes between a fresh and a healed MI [**17**]. It should be emphasized that other conditions such as acute myocarditis or transplant rejection may equally present focal or diffuse myocardial edema [**18**]. ***T2–weighted MR sequences***, equipped with inversion techniques to null the signal of fat and blood (will appear dark) (T2–weighted short–inversion–time inversion–recovery, ***T2w–STIR MRI***, “triple inversion-recovery sequences”), are now most commonly used for edema imaging [**19**].

### Contrast Enhanced MRI

Up to date, the paramagnetic gadolinium–chelated contrast agents ***(Gd)***, mainly Gd– diethylenetriamine pentaacetic acid ***(DPTA)***, are the only licensed group of paramagnetic contrast agents for cardiac imaging routine use. Gd contrast agents have been extensively evaluated for MI imaging in both the acute and chronic setting [**20, 21**]. This small molecule diffuses rapidly after intravenous injection into the interstitial space (strictly extracellular), and is eliminated by renal clearance. Due to differences in pharmacokinetics and distribution volume between normal and infarcted/scarred myocardium, the latter appears bright (enhanced) compared to normal myocardium which appears dark.

Gadolinium contrast agents are not infarct specific; as such, myocardial enhancement is non-specific, while the location and pattern of enhancement give important information regarding the underlying etiology.

Currently, in the routine clinical setting, MI imaging after Gd administration is done by an ***inversion–recovery T1–weighted sequence***, which achieves an increased contrast between normal and pathological tissue (dark vs. bright). Using this technique, infarcted myocardial tissue weighting as low as 1 gram can be visualized (vs. 10 grams with SPECT) like, for example, in papillary muscle necrosis or peri–procedural myocardial damage [**22**].

Administration of Gd contrast agents in ***acute MI*** results in a time–varying infarct enhancement. Infarct–related factors and contrast agents or MRI sequences characteristics contribute to the “observed” enhancement. Among the infarct related factors a hyperemic response after reperfusion (increased delivery of Gd) and an increased myocardial distribution volume due to interstitial edema and myocyte disruption (intracellular diffusion of contrast agent) will increase Gd concentration in the infarcted area, while an incomplete coronary recanalization with a poor collateral circulation and the presence of MVO will act the opposite [**23–25**].

Ce–MRI is a robust, well–validated and accurate tool to depict myocardial necrosis in the acute setting of MI. Following administration of contrast material, infarcted myocardium is visible as a time–varying enhancement (bright) distinguishable from normal myocardium (dark). However, one should comprehend that extracellular contrast agents are neither specific nor avid for myocardial necrosis.

**Fig. 4:**
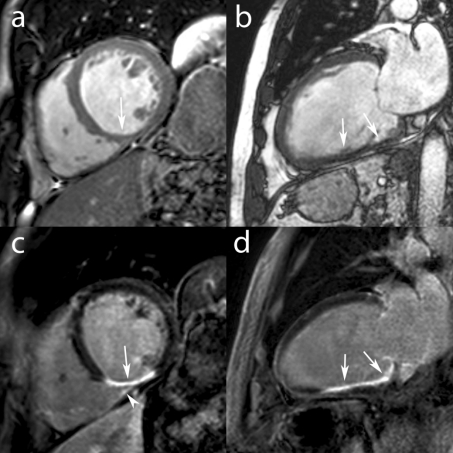
MRI study in a 58 year old male recently diagnosed with dilated cardiomyopathy (LV EF 20%). Still midventricular short-axis (**a**) and vertical long-axis (**b**) images of cine MRI show a remodeled, dilated LV and inferior wall thinning (*arrows*, **a,b**). Late ce-MRI in the same imaging planes show transmural enhancement of the basal and mid inferior wall (**c,d**) and of the mid inferomedial RV suggesting an old inferior MI with RV involvement (*arrow head*). Coronary angiography showed trivessel disease with RCA occlusion.

It is proven that in ***chronic CAD*** patients, myocardial enhancement in areas of dysfunctional myocardium corresponds closely to fixed defects on thallium SPECT, and areas of flow–metabolism matched defects on FDG–PET scans, histologically representing scarred or fibrotic tissue [**26,27**]. This technique is nowadays routinely used to depict infarct–related myocardial scarring and is helpful to differentiate dilated cardiomyopathy from LV dysfunction related to CAD, and to predict functional recovery post–coronary revascularization [**13,28**] (**Fig. 4**).

### Stable Coronary Artery Disease

As already mentioned above, cardiac MRI offers a comprehensive non–invasive imaging approach to the broad chronic CAD spectrum patients, by being able to combine both ***anatomic and ischemic imaging (rest and stress)***. Due to long acquisition times and lack of robustness, clinical use of cardiac MRI for non–invasive coronary plaque detection is nowadays of limited value. Despite this, substantial progress has been made in myocardial ischemia assessment, making this technique an attractive and valuable alternative to stress echocardiography and radionuclide pharmacological stress perfusion imaging (i.e. SPECT imaging).

Compared to stress echocardiography, MRI provides reliable image quality not limited by the adequacy of the acoustic window. Per stress level, images can be easily acquired in standardized (repeatable) planes, and easily compared on an off–line workstation reducing operator–dependency.

Compared to SPECT, MRI is able to give similar information about myocardial perfusion with superior spatial resolution, no radiation exposure, and in a single examination. Moreover, the superior spatial resolution of MRI permits to visualize smaller, subendocardially located perfusion defects not infrequently missed by SPECT [**29**].

### Stress Perfusion Imaging

MRI perfusion studies use the “first pass” of an intravenously injected Gd contrast agent during administration of a vasodilator (i.e. adenosine or dipyridamole) to depict hemodynamically significant coronary artery stenosis (**Fig. 5**).

**Figure:**
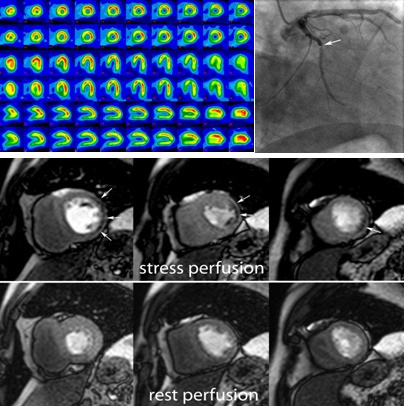
**Fig. 5**MRI study (rest and stress perfusion) in a 52 year old male with stable angina. Rest and stress (*) SPECT were negative, showing no induced perfusion defects (**a**). Midventricular short-axis cardiac MRI images (**b**) of first pass perfusion during rest (**bottom**) show no perfusion defects while during dipyridamole vasodilatatory stress (**top**) show a subendocardial perfusion defect in the lateral wall (*arrows*). Coronary angiography (**c**) confirmed a significant (90%) stenosis of the first lateral branch of Cx (*arrow*, vessel larger than the Cx).

At present this technique has been well validated, showing similar or better accuracies when compared to routinely used techniques such SPECT imaging [**29–31**]. In a meta–analysis by Nandalur et al. including 24 studies (1516 patients, high disease prevalence), perfusion imaging demonstrated a sensitivity of 91% and a specificity of 81% [**32**].

Regarding data analysis, in daily clinical practice stress–induced perfusion defects are usually visually assessed by looking at the presence of subendocardial or transmural hypo–intense (dark) rim during the ***“first–pass” of contrast*** in one or more coronary artery perfusion territories. Semi–quantitative or quantitative methods have become available mainly for research purposes. A relatively simple semi–quantitative method that has been validated against coronary flow reserve measurements is the assessment of the myocardial perfusion reserve (MPR) or MPR index. MPR index is defined as the ratio of regional myocardial blood flow after induced vasodilatation to that under resting conditions [**30,33**]. For example, Rieber et al., found that by using fractional flow reserve in addition to the degree of stenosis on coronary angiography as reference modality, a MPR index cut–off value of 1.5 was able to distinguish between hemodynamically relevant and non–relevant coronary lesions with a sensitivity of 88% and specificity of 90% [**34**]. After PCI with stent implantation or CABG surgery, a significant improvement in MPR index was reported, thus providing information on the success of interventional revascularization procedures [**35**].

It must be noted that first–pass perfusion is prone to dark–rim artifacts (dark ring at the subendocardial border) and these artifacts may interfere with depiction of subendocardial ischemia.

### Stress Function Imaging

Although it is possible to perform exercise testing in an MR environment using a specific MR–compatible ergometer (supine bicycle), functional stress testing is usually performed during dobutamine (± atropine) administration [**36**]. Similar to dobutamine stress echocardiography, a stepwise dose increment of dobutamine is used in order to differentiate the precise nature of the ischemic substrate, i.e., stunned, ischemic, hibernated, necrotic/scarred myocardium [**37,38**].

***MRI stress function imaging protocol*** is similar to stress echocardiography; acquisition is started in resting conditions with cine MR images in a set of standardized imaging planes through the ventricles (usually short axis, horizontal and vertical long–axis). This approach allows the evaluation of regional contractility in all segments of the left ventricle. In addition a single breath–hold 3D cine MRI sequence encompassing the entire left ventricle is performed, allowing the calculation of LV volumes and ejection fraction. Next, dobutamine infusion is started at low dose (5 μg/kg per minute) and increased stepwise by 100% up to 40μg/kg per minute for ischemia testing and up to 20μg/kg per minute for viability studies. The same images are acquired 3 minutes after the initiation of a new dose. The images can be transferred immediately after completion of a step to an off–line workstation and analyzed for new or worsening wall motion abnormalities (WMA). A peak stress first pass myocardial perfusion study can also be performed and it has shown value particularly in patients with concentric LV hypertrophy and in non–ischemic patients with stress inducible left bundle branch block, in which wall motion analysis is difficult.

With respect to adverse effects, studies have shown that high–dose functional stress imaging in an MR environment can be considered as safe and feasible in patients with suspected or known CAD [**39**].

The advantage of stress function MRI over echocardiography rests in superior image quality, with high spatial and temporal resolution and better inter– and intra–observer agreement [**40**]. Two studies have demonstrated the higher accuracy (86.0% vs. 72.7%) of high–dose stress dobutamine MRI compared to high–dose dobutamine stress echocardiography in detecting patients with significant CAD [**41,42**]. Patients with poor acoustic windows benefit the most from MRI stress testing.

**Fig. 6:**
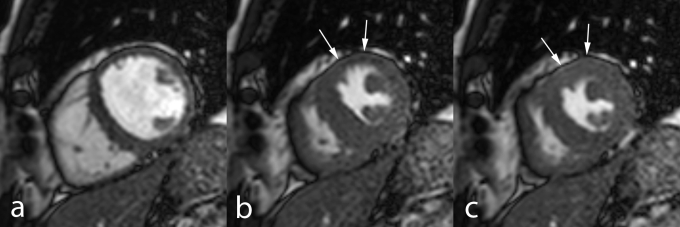
MRI study (dobutamine–atropine stress) in 60–year–old man. Midventricular cardiac short–axis cine MRI at end–diastole (**a**), end–systole (**b**), and isovolumic relaxation (**c**). At maximal stress, during systole (**b**), the ischemic myocardium in the anterior wall is akinetic (*arrows*). However, during isovolumic relaxation (**c**) while the non–ischemic regions start to relax, myocardial thickening can be is seen in the anterior LV wall (*arrows*), i.e. post–systolic contraction of the ischemic myocardium. Coronary angiography showed significant stenosis in the proximal LAD coronary artery.

The most commonly used approach in ischemia functional stress studies is ***visual analysis of new or worsening WMA’s*** using a high–dose dobutamine/atropine regimen and a 16 (or 17) segment model [**12**] (**Fig. 6**).

This yields good sensitivities (83–96%) and specificities (80–100%) for detection of significant CAD [**43**].

### Acute Coronary Syndromes

The diagnostic tools available at the moment of presentation of an acute coronary syndrome: blood biomarkers, electrocardiography and echocardiography, only provide a partial insight into the complex, evolving processes in the jeopardized myocardium. Cardiac MRI allows an accurate appraisal of the myocardium in the first hours after the onset of chest pain providing substantial complementary information to standard imaging modalities.

### Characterization of the Jeopardized Myocardium

***The myocardium (area) at risk*** is a major determinant of infarct size, because it predicts the maximal area of myocardium that is at risk for necrosis. It is known that the anatomical site of occlusion of the coronary artery does not reliably predict the size of the area at risk [**44**]. Although angiographic criteria were assessed to estimate more accurately the jeopardized myocardium, for example by using the Bypass Angioplasty Revascularization Investigation Myocardial Salvage Index (BARI), there is need for imaging techniques that can directly and accurately depict this area at risk [**45,46**].

***Edema imaging by T2-weighted sequences*** is at present one of the most appealing techniques to non–invasively assess the myocardium at risk [**47**]. This can be determined retrospectively, i.e. post–reperfusion, by T2–weighted edema imaging, with abnormalities present at least one week after the acute event [**16**]. Abdel–Aty et al., recently showed that edema imaging depicts acute ischemic injury within the first 30 minutes after onset of ischemia, before the onset of irreversible myocardial injury (i.e. troponin elevation, late Gd enhancement) [**47**]. Since the development of myocardial edema is an early feature in acute coronary syndromes, adding T2–weighted imaging to the MRI exam in unstable angina or MI patients, may serve as a very useful diagnostic marker in the emergency department [**48**].

**Fig. 7:**
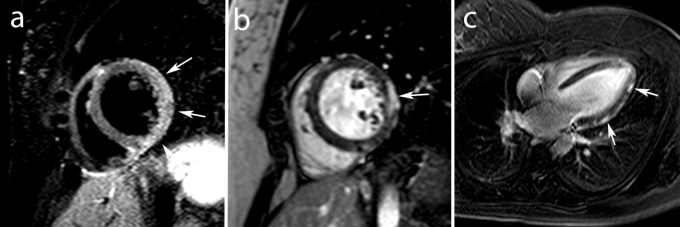
MRI study in an 18 year old male with acute chest pain in which acute myocarditis was diagnosed. Mid ventricular short axis T2–weighted STIR image shows edema (increased signal intensities) in the lateral wall (*arrows*, **a**) and short and vertical long–axis ce–MRI show typical subepicardial enhancement in the lateral wall (*arrows*, **b,c**).

As mentioned above, edema imaging is not MI specific and in the acute setting of cardiac–related chest pain, patients with Tako–Tsubo cardiomyopathy and acute myocarditis may show also myocardial edema (**Fig. 7**).

Ce–MRI is a valuable tool for detailed ***infarct imaging***. Myocardial enhancement after Gd contrast administration within the jeopardized myocardium reflects mainly irreversibly damaged tissue that will be lost [**23,24,49**]. Myocardial contrast wash–in and wash–out are dynamic processes, so one of the most important factors in imaging post contrast administration is timing [**50**]. The optimal time window for infarct imaging being between 10 to 25 minutes post contrast administration, we refer to it as ***late ce–MRI***opposed to ***early ce–MRI*** which is generally used to depict ***MVO***. Moreover, the extent of enhancement is significantly influenced by the time between time of infarct and imaging, with a significant decrease in extent of enhancement between day 1 and day 7 post–infarction [**24,49**].

***Myocardial salvage*** by early reperfusion therapy can be objectively assessed by relating the extent of necrosis to the extent of myocardium at risk [**51**]. Myocardial salvage can be determined by SPECT, but this technique suffers from several limitations. Similar information are non–invasively obtained using a combination of ***edema imaging and late ce–MRI***. The myocardium at risk exceeds the irreversible damaged myocardium, but both are closely related [**52,53**]. Typically, edema involves the entire width of the myocardial wall, whereas the transmural spread of necrosis is variable. Francone et al. showed in an MRI study that increasing ischemia time does not affect the extent of myocardium at risk but results in an increasing infarct size and subsequently decreased myocardial salvage [**54**]. The amount of salvaged myocardium is markedly reduced after 90 min of coronary occlusion, while early mechanical reperfusion and maintenance of antegrade or collateral flow independently preserves myocardial salvage primarily through a reduction of infarct transmurality [**54,55**]. Myocardial salvage is independently associated with early ST–segment resolution, and is an independent predictor of adverse LV remodeling and major cardiac events [**51–53**]. Early reperfusion can result in ***aborted infarction***, thus achieving the ultimate myocardial salvage [**56**]. Although the diagnosis of aborted MI is based on complete resolution of initial ST–segment elevation and lack of significant increase in cardiac enzymes, a combined ***edema imaging*** and ***ce–MRI*** approach can help in visualizing aborted MI.

### Assessment of Myocardial Infarction Severity

***Infarct size*** is a crucial determinant of adverse LV remodeling. Thus, estimation of the amount of irreversibly lost myocardium is imperative to assess infarct severity and has short and long term prognostic value [**57**]. Late ce–MRI is a well–validated, accurate and reproducible tool for sizing acute, healing and healed infarcts [**58,59**]. Due to its high spatial resolution, enabling depiction of small MI’s, late ce–MRI is considered the reference imaging modality for infarct sizing and is increasingly being used to determine the relationship between infarct size, ventricular remodeling and patient outcome [**26,58,60,61**]. As for acute MI patients, in patients with healed or unrecognized myocardial infarctions, the presence and extent of scarred tissue on ce–MRI shows strong and independent association with major adverse cardiac events and cardiac mortality [**62–64**].

***Infarct transmurality*** is another important parameter to be taken into consideration when assessing infarct severity. As it has been shown, early restoration of myocardial perfusion might limit the transmural wave of necrosis and salvage the jeopardized, viable myocardium in the outer, subepicardial layers [**54**]. Ce–MRI enables the accurate determination of infarct transmurality and is superior to other techniques such as SPECT imaging [**24,26**]. Infarct transmurality can be expressed visually using a semi–quantitative score (1: 0%, 2: 0–25%, 3: 26–50%, 4: 51–75%, 5: 76–100% of total wall thickness) or using an automated quantification approach [**65,66**]. Several ce–MRI based studies have shown that increased infarct transmurality is related to lack of inotropic reserve and impaired recovery of contractile function, and is associated with more pronounced post–infarct wall thinning, aneurysm formation and adverse ventricular remodeling [**49,67–69**]. In patients with healed infarcts and ventricular dysfunction, scar transmurality as determined by ce–MRI, predicts functional recovery post–revascularization and patient survival [**13,63**].

***Microvascular obstruction or No–Reflow***. Even with early, successful and sustained restoration of coronary flow in the culprit coronary artery, between 5 and 50% of STEMI patients show lack of restoration of blood flow at myocardial level. This ***no–reflow*** phenomenon is secondary to ***microvascular obstruction (MVO)*** and has a complex, multifactorial pathogenesis including: distal embolization, ischemia–reperfusion injury, and individual coronary microcirculation predisposition to injury [**4,70**]. No reflow increases with the duration of ischemia time, is related to more severe myocardial damage and is independently associated with lack of functional recovery, adverse ventricular remodeling and worse patient outcome [**54,68,69,71–74**].

No–reflow can be assessed during primary PCI with Thrombolysis In Myocardial Infarction (TIMI) flow grade and myocardial blush grade (MBG) post–reperfusion, by ST–segment elevation resolution early post–reperfusion, and can be better quantified by noninvasive imaging techniques such as myocardial contrast echocardiography and ce–MRI [**70**]. Ce–MRI has become the preferential technique for MVO depiction and has been validated against pathologic studies using specific staining (thioflavin–S). On ce–MRI no reflow is visualized as a central or subepicardial, hypo–intense, dark region within the enhanced, bright area of infarcted myocardium and is clearly distinguishable from completely reperfused infarcts presenting as homogeneous enhancement (**Fig. 8**).

**Figure:**
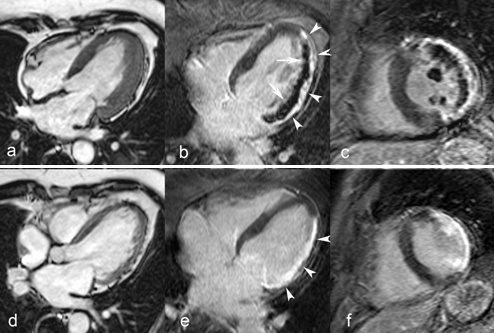
**Fig. 8**Extensive lateral MI in 68 year–old man studied 1 week and 4 months after the acute event. Proximal Cx coronary artery occlusion treated by primary PCI but complicated by no reflow. Cine MRI in horizontal long–axis shows thickened LV lateral wall due to myocardial edema and inflammation in the acute phase (**a**), followed by marked wall thinning at 4 months (**d**). Horizontal long–axis and midventricular short–axis late ce–MRI show an extensive subendocardial no reflow zone at 1 week (*arrows* **b,c**) and transmural enhancement of the lateral wall (*arrowheads*, **b, c, e, f**). Both papillary muscles are involved (no reflow at 1 week and enhancement at 4 months). At follow–up strong, homogeneous enhancement of the scarred myocardium (*rrowheads*, **e**).

Studies using early ce–MRI have shown a progressive increase in size for the hypo–intense region corresponding to no reflow (up to 3–fold) during the first 48 hours, suggesting progressive microvascular damage after reperfusion [**75**]. Beyond 2 days, the progress causing no–reflow expansion most likely stabilizes and persists at least till 9 days post–infarction [**76**].

To appreciate the extent of MVO, ***early ce–MRI*** should be performed, i.e. the first 1–4 minutes, following intravenous contrast administration. ***First–pass perfusion imaging*** and ***late ce–MRI*** can also be used for MVO imaging. On first–pass perfusion MVO is recognized by the presence of hypo–enhanced, dark region (perfusion defect) despite successful restoration of coronary flow in the infarct–related artery. Using ***late ce–MRI*** MVO is visualized in the same way as for ***early ce–MRI***, i.e. dark areas within the bright enhanced infarct area, but usually is smaller in size in the late sequences. For example, in 52 reperfused ST–elevation MI’s, we found a reduction in number (32 versus 27 patients) and spatial extent of no–reflow (36±25% versus 16±14% late enhancement extent) between early and late ce–MRI [**77**].

Reperfusion injury and microvascular damage, in their most severe form, can be associated with significant interstitial extravasation of red blood cells, i.e. ***post–reperfusion intramyocardial hemorrhage *** [**78**]. Hemorrhagic myocardium has a typical heterogeneous appearance on ***T2–weighted MRI (edema imaging)*** due to formation of hemoglobin breakdown products containing iron and their paramagnetic effects. This heterogeneous appearance is visualized as a hypo–intense core with a peripheral hyperintense rim, while, non–hemorrhagic infarcts present as areas with homogeneously increased signal intensity in the myocardium at risk [**79**]. In a study of 98 patients with reperfused STEMI’s, we found intra–myocardial hemorrhage in 25% of patients [**80**]. Patients with hemorrhagic infarcts had a lower pre–TIMI flow, higher cardiac enzymes, larger infarct size and greater infarct transmurality, larger no–reflow zone, and a lower myocardial salvage index. Intra–myocardial hemorrhage was an independent predictor of adverse LV remodeling regardless of the initial infarct size. It should be emphasized that intra–myocardial hemorrhage has a close and intricate relation with MVO. On the one hand, the degree of microvascular injury underlies extravasation of red blood cells and on the other hand, intra–myocardial hemorrhage may worsen tissue turgor and aggravate the severity and extent of MVO.

***Right ventricular (RV) infarction***, usually an expression of a biventricular acute MI is characterized by a faster and better functional recovery than LV due to a more favourable oxygen demand/supply profile for the former [**81**]. Lack of RV recovery, however, is associated with persistent hemodynamic compromise and high mortality rate [**82**]. MRI is increasingly being used to study RV ischemic injury in MI patients, though assessment of reversible and irreversible ischemic injury in a thin and trabeculated RV wall is challenging (**Fig. 4**). Despite some limitations, MRI has contributed not only to a better depiction but also to a better understanding of RV ischemic injury. It has become clear that current established techniques for RV infarct detection, such as clinical investigation, ECG with right precordial leads, and echocardiography underestimate the true incidence of RV ischemic injury in acute inferior LV infarcts, with RV inferior wall enhancement in approximately 50% of patients. Moreover, RV ischemic injury is not limited to inferior infarcts but is often found in anterior LV infarcts as well [**83**]. Taking into account that up to 30% of the RV free wall is perfused by the LAD coronary artery, a considerable portion of the RV myocardium is at risk in LAD infarcts. Fortunately, the RV myocardial salvage post–reperfusion is large and the resultant RV infarct size small [**83,84**]. In a recent study we found at 4–month follow–up post–infarction a significant decrease in extent and frequency of RV myocardial enhancement [**83**]. Potential explanations include volume shrinkage due to infarct healing but other mechanisms such as acute, temporary enhancement of the reversibly injured portion of the jeopardized myocardium should be equally considered.

### In Vivo Morphologic Validation of ECG using Contrast Enhanced MRI

In the past, a great deal of knowledge about ECG changes in acute coronary syndromes and IHD was based on pathologic correlations in animal work and autopsy studies. Ce–MRI has allowed a reassessment and reinterpretation of our common understanding of the ECG [**65**].

First, regarding Q waves and transmurality, several MRI studies have shown that the primary determinant of the presence of a Q–wave is the total size of the underlying infarction rather than its transmural extent [**85–87**].

Second, ECG–derived estimates of infarct size correlate at best, only modestly with ce–MRI, overestimating small infarcts and underestimating large infarcts [**88**][88]. Importantly, the lateral LV territories are electrically silent and therefore may present with little ECG alteration [**89**]. Recently, a multi–specialist team has defined a new terminology of the LV walls and proposed a new ECG classification of Q–wave myocardial infarction based on cardiac MRI and angiographic correlations [**90**].

### Patterns of Enhancement Depending on Infarct Location

As already mentioned above, myocardial enhancement after ce–MRI is non-specific for MI. Two important features attribute with a higher degree of certainty the pattern of ce–MRI enhancement to IHD.

First, regardless of the MI location, the pattern of enhancement always presents as spreading outward from the endocardium. Whether the infarction itself is subendocardial or transmural, the subendocardium is always involved.

Second, the infarction is always located within the anatomical territory supplied by one coronary artery, and the intensity of abnormalities increases towards the distal perfusion territory of that coronary artery.

The use of these two criteria allows the physician to differentiate between MI and enhancement seen in other diseases such as in myocarditis, which is known to present a more diffuse of multifocal enhancement pattern, often subepicardially located and not infrequently accompanied by pericardial involvement, i.e. pericardial enhancement (**Fig. 7**).

In inferior infarctions, the enhancement is usually located in the basal and mid–ventricular inferior LV wall and may extend towards the lateral LV wall as well towards the RV inferior wall. Lateral MI’s are typically located in the lateral LV wall and sometimes may be extensive involving the entire lateral wall. Anterior infarcts are variable in size and typically involve the anterior or anteroseptal wall showing a variable extension to the septum and anterolateral wall. Longitudinally they usually involve the mid – and apical LV portion and LV apex but may extend toward the basal LV portion. Smaller sized infarctions, often at atypical locations (e.g. mid–septum), can be seen when coronary artery side branches are involved (**Fig. 9**).

**Figure:**
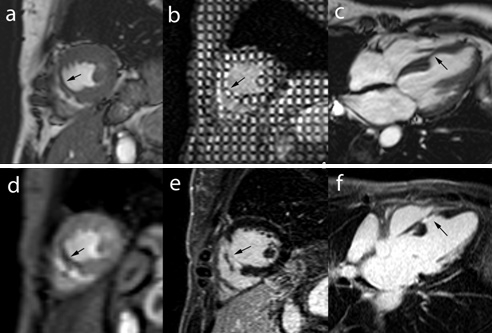
**Fig. 9**MRI study in a 47 year–old female with LV septal wall motion abnormalities (incidental findings) on echocardiography probably due to an embolic occlusion of a septal LAD coronary artery branch. Midventricular short–axis and horizontal long–axis cine MRI (**a,c**) and short–axis tagging MRI (**b**) show marked mid septal wall thinning with dyskinetic wall motion (*arrows*). First–pass perfusion MRI (**d**) and late ce–MRI (**e,f**) show a perfusion defect (**d**) and transmural enhancement (e, f) within the thinned myocardium (*arrows*).

### Infarct-related Complications

**Fig. 10:**
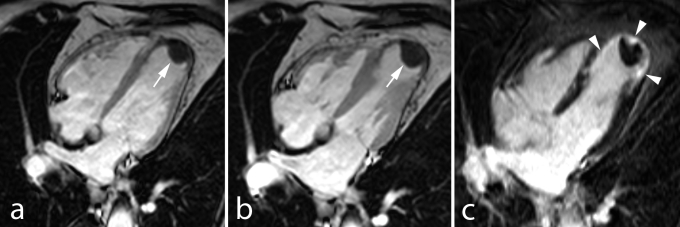
Old anterior MI in 75 year old man presenting with heart failure symptoms. Horizontal long–axis cine MRI (**a**) and early (**b**) ce–MRI show apical aneurysm with large typically hypoenhanced thrombus (*arrows*, **a,b**). Late ce–MRI in the same plane shows apical transmural enhancement (*arrowheads*, **c**).

Cardiac MRI also represents a valuable tool in the diagnosis of complications related to MI, such as aneurysm formation, thrombus formation, valve leakage, associated pericardial effusion or post–infarction pericarditis. This technique is useful to depict and differentiate true from ***pseudo ventricular aneurysms***. Besides differences in anatomy between the two, false aneurysms invariably show pericardial enhancement [**91**]. ***Ventricular thrombi*** are easily missed on transthoracic echocardiography especially when located in the LV apex or when trapped within the endocardial trabeculations. Using a combination of ce–MRI and cine MRI, ventricular thrombi are well demonstrated and differentiated from slow flow [**92**]. Thrombi are best visualized on early ce–MRI sequences and they appear as intracavitary, hypo–intense filling–defects [**93**] (**Fig. 10**).

High–spatial resolution 3D ce–MRI techniques offer improved detection for ***papillary muscle infarction*** as a substrate for mitral valve regurgitation (**Fig. 8**) [**94**]. MI associated ***pericarditis*** can also be depicted by late ce–MRI showing bright, enhanced pericardium with or without pericardial fluid [**95**]. MRI may be occasionally helpful in detecting rare complications such as ventricular septal defects or intramural dissecting myocardial hemorrhage [**96**].

**Fig. 11:**
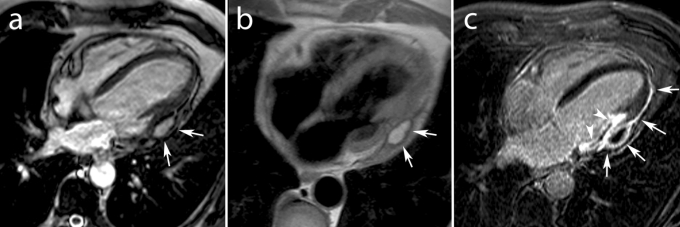
MRI study in a 66 year asymptomatic male with an old history of severe thoracic trauma (car accident) in which routine echocardiography showed an abnormal structure over the LV lateral wall. Horizontal long–axis cine (**a**) and T1–weighted (**b**) MRI show a well delineated, homogeneous and hyperintense pericardial mass (*arrows*, **a,b**). Late ce–MRI in the same imaging plane shows pericardial enhancement with the hypoenhancement of the mass (*arrows*, **c**) and adjacent transmural lateral myocardial wall enhancement (*arrowheads*, **c**). The combination of hyperintense signal on T1w with hypoenhancement on late ce–MRI is typical for old blood supporting the diagnosis of posttraumatic pericardial hematoma with secondary extrinsic compression and scarring on the adjacent LV wall.

Looking at treatment–related complications, ce–MRI can depict ***peri–procedural necrosis*** after successful PCI and coronary bypass surgery and can help for a better understanding of its underlying mechanisms [**97,98**]. MRI yields promise to accurately depict subtle myocardial damage related to other procedures like trauma, chest compression and external defibrillation [**99**] (**Fig. 11**).

### Chronic Ischemic Cardiomyopathy and myocardial viability assessment

Patients with ***chronic ischemic cardiomyopathy*** have single or multivessel CAD and dilated, dysfunctional ventricles containing a mixture of different ischemic substrates ((i.e. stunned, ischemic, hibernating, necrotic, scarred myocardium) in the same/different perfusion territory. Viability assessment is of utmost importance in deciding whether a patient may benefit from a revascularization procedure, with the aim of improving regional and global ventricular function, negative remodeling, symptoms, exercise capacity and long term prognosis [**100**].

Among the different tools available for ***viability assessment***, MRI has emerged as one of the preferential techniques by combining information about wall thickness and myocardial contractility reserve, used also by echocardiography, with the noninvasive visualization of even subtle amounts of myocardial scar formation [**26,101**]. Viability imaging with MRI is only part of a more comprehensive approach which involves evaluation of ventricular volumes and global function, visualization and quantification of associated valvular heart disease (i.e. mitral regurgitation), and depiction of complications such as ventricular aneurysms or thrombus formation [**92**].

A first approach is the measurement of segmental end–diastolic wall thickness (EDWT). The degree of wall thinning secondary to MI healing and scar formation is related to the degree of infarct transmurality. This approach appears very sensitive (95%), but not specific (41%) for prediction of functional recovery, indicating that thinned myocardium (< 6mm) has a low likelihood to improve function after revascularization and accurately reflects scar tissue. While a substantial percentage of segments with preserved wall thickness do not improve in function following revascularization, probably due to the presence of subendocardial infarctions, thinned myocardial segments may undergo a process of “reverse remodeling” after successful myocardial revascularization with recovery of function and regain in regional wall thickness [**100,102**].

A second approach is the ***contractile reserve assessment*** during low dose dobutamine stress. In patients with chronic LV dysfunction, hibernating myocardial segments typically show a ***“biphasic response”*** with improved contractility during low–dose (5–10 μg/kg per minute) dobutamine infusion, followed by a worsening of function [**103**]. Dobutamine stress MRI for viability testing has good specificity (83%) but moderate sensitivity (74%), values that are in line with those of dobutamine echocardiography [**100,102**].

***Contrast–enhanced MRI***, the third approach, detects scar tissue but not viability. In brief, the likelihood of improvement in regional contractility after revascularization decreases progressively as the transmural extent of enhancement before revascularization increases. 78% of dysfunctional segments without enhancement showed improved contractility postrevascularization, compared to 2% of dysfunctional segments with scar tissue extending >75% of the LV wall in a study by Kaandorp et al. This technique had an excellent sensitivity (95%) but a low specificity (45%) [**104**]. The low specificity is related to subendocardial infarcts in which it is unclear whether the non–enhanced subepicardial myocardium contains normal, viable or jeopardized myocardium.

Because none of the above approaches is perfect in neither predicting nor excluding functional recovery after revascularization, an ***integrated use of MRI techniques*** may improve diagnostic accuracy. Kaandorp et al. proposed to start with ce–MRI to depict myocardial scarring and determine the transmural extent. Then, in segments with an ***intermediate extent of scar tissue*** (transmurality 25–50%), in which the likelihood of recovery is uncertain, ***dobutamine stress MRI*** may be used to differentiate between those with versus those without contractility reserve [**104**].

### Prognosis Assessment

Any clinical perspective about an imaging technique should involve its role in patient prognosis assessment and management. There is growing evidence that cardiac MRI is an increasingly important tool in the management of cardiovascular disease [**105**].

In patients with ***suspected CAD***, a negative stress perfusion MRI study yields a high negative predictive value for future major adverse cardiac events; whereas stress perfusion defects or dobutamine stress induced WMA’s predict subsequent cardiac events [**64,106,107**]. Moreover, in patients without a history of MI but with clinical suspicion of CAD, the presence of myocardial enhancement even in small amounts, reflecting ischemia-related myocardial scarring, carries an increased risk for future major adverse cardiac events [**62,64**].

In low to intermediate risk ***unstable angina*** patients, adenosine stress perfusion MRI is a more accurate predictor of future cardiac events than traditional cardiac risk factors [**108**]. Moving forward to acute MI, in ***STEMI*** patients, MRI has become the reference technique for infarct sizing. Moreover, because of its unique capability for characterization of the infarct and jeopardized myocardium MRI is a valuable tool in the assessment of other factors associated with a poor prognosis, besides infarct size) [**8,11,57,109**]. These include MVO, post–reperfusion myocardial hemorrhage, the peri–infarct area, and myocardial salvage [**51,53,73,76,80,110**]. Also in patients with ***NSTEMI***, persistent MVO is an independent predictor of major cardiac events [**111**].

In ***ischemic cardiomyopathy*** patients with severely reduced LVEF, the extent of myocardial enhancement is associated with increased mortality or the need for cardiac transplantation. In a study involving 857 consecutive patients with and without LV dysfunction and a median follow–up of 4.4 years, myocardial enhancement using a myocardial scar index (i.e. sum of transmurality scores of all 17 segments divided by 17) was a strong and independent predictor of all cause mortality/cardiac transplantation [**63**].

**Sources of Funding**: none.

**Disclosures**: none.
